# The concept of the mobilized domain: how it can explain and predict the forces exerted by a cohesive granular avalanche on an obstacle

**DOI:** 10.1007/s10035-021-01196-1

**Published:** 2022-02-11

**Authors:** M. L. Kyburz, B. Sovilla, J. Gaume, C. Ancey

**Affiliations:** 1 WSL Institute for Snow and Avalanche Research SLF, Davos, Switzerland; 2 Environmental Hydraulics Laboratory, École Polytechnique Fédérale de Lausanne, Lausanne, Switzerland; 3 Snow and Avalanche Simulation Laboratory SLAB, École Polytechnique Fédérale de Lausanne, Lausanne, Switzerland

**Keywords:** Cohesive granular flow, Impact pressure calculation, Impact pressure on obstacles, Mobilized domain

## Abstract

**Abstract:**

The calculation of the impact pressure on obstacles in granular flows is a fundamental issue of practical relevance, e.g. for snow avalanches impacting obstacles. Previous research shows that the load on the obstacle builds up, due to the formation of force chains originating from the obstacle and extending into the granular material. This leads to the formation of a mobilized domain, wherein the flow is influenced by the presence of the obstacle. To identify the link between the physical mobilized domain properties and the pressure exerted on obstacles, we simulate subcritical cohesionless and cohesive avalanches of soft particles past obstacles with circular, rectangular or triangular cross-section using the Discrete Element Method. Our results show that the impact pressure decreases non-linearly with increasing obstacle width, regardless of the obstacle’s cross-section. While the mobilized domain size is proportional to the obstacle width, the pressure decrease with increasing width originates from the jammed material inside the mobilized domain. We provide evidence that the compression inside the mobilized domain governs the pressure build-up for cohesionless subcritical granular flows. In the cohesive case, the stress transmission in the compressed mobilized domain is further enhanced, causing a pressure increase compared with the cohesionless case. Considering a kinetic and a gravitational contribution, we are able to calculate the impact pressure based on the properties of the mobilized domain. The equations used for the pressure calculation in this article may be useful in future predictive pressure calculations based on mobilized domain properties.

**Graphic Abstract:**

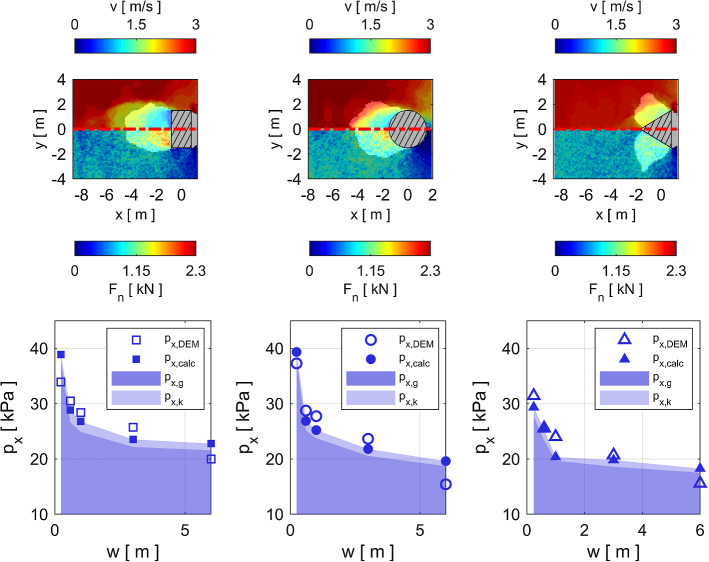

**Supplementary Information:**

The online version contains supplementary material available at 10.1007/s10035-021-01196-1.

## Introduction

The question of how bodies and granular materials moving relative to each other interact and create interaction forces is of fundamental interest in fluid dynamics and for the rheology of granular flows [1]. The understanding of the interaction processes is also important for manifold practical applications, such as the transportation of particles [2], the impact of objects in granular beds [3], and the structural design of obstacles subjected to geophysical flows [4–6]. Our research is motivated by the need to understand the relevant processes of cohesive granular snow avalanches interacting with obstacles in the flow path. Endangered obstacles are mostly located in or close to settlement areas or traffic routes. When avalanches reach these obstacles they are often in the run-out phase and therefore moving relatively slowly. Hence, in our research we focus on a cohesionless and a cohesive granular flow characterized by a subcritical Froude number $Fr<1$ interacting with an obstacle. The Froude number is defined as $Fr=v/{\sqrt{g\,h}}$, where *v* is the undisturbed velocity of the granular material, *g* is the gravitational acceleration, and *h* is the height of the undisturbed flow surface above the ground. For subcritical flows the force exerted on an obstacle by the granular flow is mostly independent of the flow velocity [2, 7–10].

Previous research shows that the force acting on the obstacle originates from force chains forming between jamming particles [11, 12]. On the particle scale, the flow–obstacle interaction dynamics are therefore governed by the coexisting formation and destruction of these force chains extending upstream of the obstacle into the flow. Cohesion is known to increase the persistence of the force chains and the contact network density [13, 14]. On the macroscopic scale, the strong force chains originating from the obstacle form a region which is referred to as the mobilized domain (MD) by some authors (e.g., [15–17]). Hence, the MD is the region in a granular flow encountering an obstacle, which experiences a significant increase in the contact forces between particles. Presumably, the macroscopic force experienced by the obstacle is therefore governed by the properties of the MD [16].

To our knowledge, the MD has only been described in a few studies, and the authors provided little information on the implications for the drag force on the obstacle [8, 16, 18]. Chehata et al. [8] suspect that the drag exerted on an obstacle is the result of compressive stresses acting on the MD. Revisiting a large number of studies on the macroscopic force on obstacles, Faug [16] proposes a phenomenological model to calculate the force, considering a kinetic, a gravitational and an apparent weight contribution.

In the present study we aim to identify the relevant interaction processes and MD properties to physically link the MD and the average impact pressure on the obstacle. In order to achieve this objective, we use the Discrete Element Method (DEM) to simulate a volume of moving granular material consisting of soft discrete particles. In our setup the moving particles interact with a static obstacle, whereat we systematically identify the MD around the obstacle. Because we focus on subcritical flows, for which the impact pressure is independent of the velocity, we arbitrarily select a flow velocity of $v=3$ m/s, corresponding to $Fr=0.61$. The granular flow interacts with obstacles with rectangular, circular and triangular cross-sections of widths between 0.24 m and 6.0 m. In order to assess the influence of the cohesion on the impact pressure, we simulate a cohesive and a cohesionless scenario for all combinations of obstacle widths and geometries. Because compressive stresses supposedly play an important role in the impact pressure [8], we perform axial compression tests with the same cohesionless and cohesive granular material to assess how the compressed material state in the MD is linked to the stress inside the material and on the obstacle.

We organize the article as follows. In Sect. 2 we describe our numerical model and the setup of our simulations. In Sect. 3 we present the results of our simulations of granular flows interacting with obstacles of various geometries. Thereafter, we discuss the presented method, the results and the method’s limitations in Sect. 4. Finally, we summarize the most important points of the paper with our conclusions in Sect. 5.

## Materials and methods

In this section we describe the methods and parameters used to simulate the interaction of granular flows and obstacles of various geometries and sizes. First, we present in detail the numerical setup and the simulation procedure. Second, we present the obstacle geometries for which we simulate the interaction with the granular flow. Third, we define the contact model, as well as the material and flow properties of the granular material used in our study. Fourth, we present axial compression tests showing how this material behaves under compressive loading. Finally, we present how we can distinguish the domain where the granular material flows freely from the domain where the flow is affected by the presence of the obstacle.

### Simulation setup and procedure

The present model is implemented in the *PFC* Discrete Element Method (DEM) software from Itasca (Minneapolis, MN, USA), which is based on the soft-contact algorithm for the interaction of discrete spherical particles [19].

**Fig. 1:**
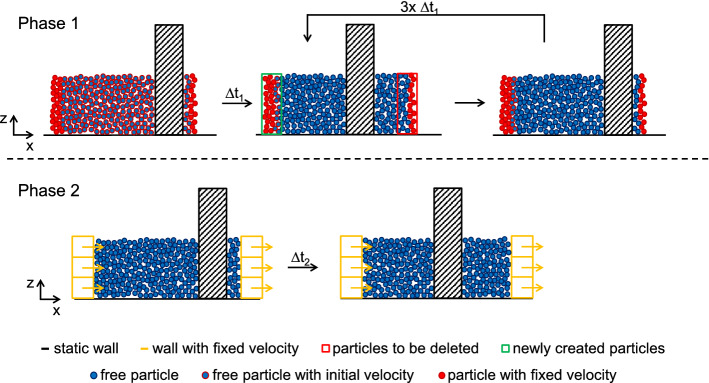
Simulation procedure in two phases. First phase (top) with continuous particle generation and deletion at the boundaries. Second phase (bottom) where the flow is imposed by the boundary walls

In this study, we simulate granular flows with and without cohesion (Sect. 2.3) interacting with obstacles of different geometries and sizes (Sect. 2.2). As input for the simulation we want to impose the same boundary velocity of the granular material independently of the properties of the granular material and the obstacle geometry. Assuming that the free boundaries of large granular avalanches are far from the obstacle, we only simulate an isolated volume confined within the surrounding granular material in the flow. We therefore impose the motion of the granular material at the up- and downstream boundaries of the isolated volume. In the streamwise *x* direction the granular material is confined between either fixed particles or boundary walls, as shown in Fig. 1. In the simulations with obstacle widths $w\le {}1.0$ m, we use a domain length of $D_x=11$ m in the *x* direction. For wider obstacles we use $D_x=22$ m. In the *y* direction transverse to the flow, the domain is limited by a periodic boundary condition and has a width of $D_y=28$ m. We check that these domain sizes are sufficient to avoid strong force chains, and thus the MD originating from the obstacle, reaching the domain boundaries.

In the vertical, *z* direction the domain is $D_z=28$ m tall. Because the granular material is subjected to gravity acting in the $-z$ direction, there is only a bottom boundary wall to confine the particles in the vertical direction.

When the flow first impacts the obstacle at the beginning of a simulation, the flowing material has yet to form the MD. Hence, to investigate how the impact pressure on an obstacle is physically linked to the flow around it, we need to obtain a continuous flow with a MD around the obstacle. In order to achieve this while minimizing the computational effort, we split the simulation procedure into two phases which we describe below. The first and the second phase are shown schematically in the upper and the lower half of Fig. 1, respectively. While the particles can flow around the obstacle in our 3D setup, Fig. 1 shows in sectional a view in the *x*-*z* plane in the middle of the flow domain.

In the first phase we establish the flow of the granular material around the obstacle. In the beginning all particles are assigned an initial or boundary velocity in the streamwise *x* direction (particles with a red outline in Fig. 1). During a simulation period $\varDelta {}t_1$ the velocity of the particles at the up- and downstream boundary (particles with a red outline and fill) is fixed at the boundary velocity in the *x* direction, and at zero in the *y* and *z* directions. The rest of the particles are free to move according to the interaction with other particles or the obstacle (particles with blue fill). Hence, the boundary particles push the freely moving particles in the flow direction past the obstacle.

After the simulation period $\varDelta {}t_1$ the simulation is paused and the fixed velocity condition is released for all particles. Particles beyond the downstream boundary are deleted (red outline). At the upstream boundary the domain is filled with newly generated particles (green outline). The newly generated particles are again assigned the initial velocity. Again, the velocities of the particles at the up- and downstream boundaries are fixed (particles with red outline and fill), while the particles further from the boundary move freely. Subsequently another period $\varDelta {}t_1$ is simulated. To develop the MD around the obstacle, we repeat this procedure three times. However, the generation of new particles in the first phase causes fluctuations in the system. Because we want to obtain a continuous force on the obstacle, we simulate a second phase where the granular material is moving continuously.

In the second phase we implement boundary walls up- and downstream of the granular material to push it continuously past the obstacle. In this configuration the fixed boundary velocity is only prescribed at the boundary walls (orange walls in Fig. 1), while all particles are moving completely freely (particles with blue outline and fill). To avoid a situation where the upstream boundary influences the mobilized domain around the obstacle, we stop the simulation after $\varDelta {}t_2$ when the boundary walls have travelled half the domain length $D_x$ in the streamwise direction.

While the impact pressures shown in Fig. 5 are calculated as the mean value of the pressure on the obstacle during the second phase (Supplementary Material S.1), for the results in Fig. 7 we consider the instantaneous impact pressure values in this phase. In Figs. 8 and 9, we report the instantaneous impact pressure values of the last time step of the simulations, as we relate the pressure to the MD properties extracted at this last time step (Sect. 2.5).

In [20] we implemented a similar setup consisting only of the second simulation phase described above. There we showed that the presented numerical procedure is able to reproduce impact pressure of snow avalanches measured in full-scale field experiments.

### Obstacle geometries

**Fig. 2:**
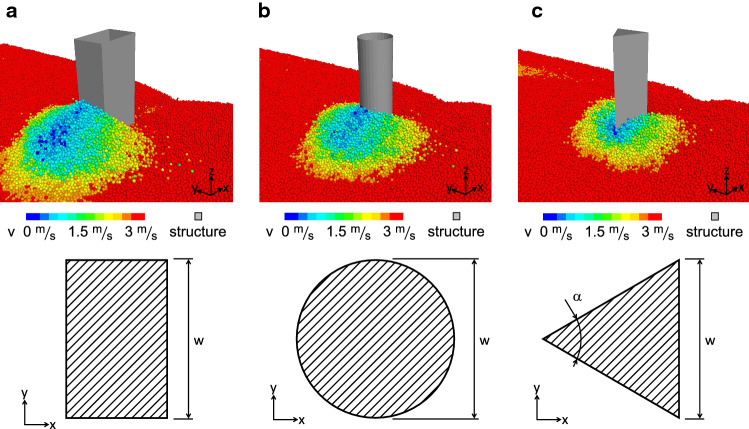
Prismatic obstacles with a rectangular (**a**), circular (**b**) or triangular cross-sections (**c**). The top row shows obstacles with $w=1$ m interacting with the granular flow. The particles are colored according to their streamwise velocity, where red corresponds to 3 m/s and blue to 0 m/s. The bottom row shows the cross-sections with the most important geometric measurements (color figure online)

To study the influence of the obstacle geometry on the MD and the impact pressure, we implement prism shaped obstacles with rectangular, circular and triangular cross-sections, as shown in Fig. 2. In all simulations the obstacles are fixed in place and are rigid, consequentially not deforming under the experienced forces. All of these prismatic obstacles have a height of 5.7 m, which prevents the granular mass from overflowing the obstacle. We consider obstacles with widths *w* of 0.24 m, 0.6 m, 1.0 m, 3.0 m and 6.0 m. We select these widths based on the dimensions of already existing obstacles measuring the impact pressure of avalanches in field experiments [21], which are 0.24 m, 0.6 m and 1 m wide. In our setup the obstacle widths are limited by our current computational resources, which do not allow the simulation of larger domains needed to avoid boundary effects for obstacles $w>6$ m.

For the rectangular cross-sections, two sides are normal and two sides are parallel to the flow direction. The sides normal to the flow are of varying width *w*, while the sides parallel to the flow are 1.6 m in all simulations. A comparison of two simulations with obstacle lengths of 0.1 m and 1.6 m in the streamwise direction shows that the pressure only deviates by 0.3 % between the two cases. Hence, we expect that using a length of 1.6 m for all rectangular obstacles does not affect the results considerably.

For the circular cross-sections the width *w* corresponds to the diameter. For the triangular cross-sections we define the angle of the wedge facing the flow as $\alpha =60^{\circ }$. Hence, the width *w* of the triangular obstacles is given by the length of the downstream side of the triangle.

The force exerted by the granular flow on the obstacle is calculated by summing the contact forces of all particles in contact with a surface. Because we simulate symmetrical obstacles and flow conditions, the force $F_y$ in the *y* direction on the obstacles is $<1\,\%$ of the total force in all simulations. The force $F_z$ in the vertical direction is $<5\,\%$ of the total force in all simulations. We therefore neglect $F_y$ and $F_z$ for the analysis of this study and only consider the force $F_x$ exerted on the obstacle in the streamwise *x* direction.

To obtain a measure of the force exerted by the granular flow that is independent from the surface area and the geometry, we define the projected impact pressure $p_x$. It is equal to the impact force in flow direction $F_x$ divided by the obstacle’s area $A_{yz}=w\,h$ facing the flow projected on the $y-z$ plane normal to the flow direction $p_x=F_x/A_{yz}$.

### Granular flow and material properties

The formulation of the Discrete Element Method and the model setup we use for this study are generic. If suitable particle and contact properties are chosen, the model can be used to simulate the interaction of a variety of cohesive granular materials and obstacles. However, in the context of snow avalanches, we choose suitable material properties for avalanche modeling. The most important contact and particle properties are summarized in Table 1. A more in-depth description of the parameter choices and the contact model can be found in [20] and its supplementary material.

In our model all particles are subjected to a gravitational acceleration of $g=9.81$ m/s$^2$ in the negative *z* direction. The mean diameter $d_p$ of the particles is 0.08 m with a polydispersity of 20 % to avoid crystallization. The particles have a density of $\rho _p=500$ kg/m$^3$ and a restitution coefficient of $e_r=0.05$. As we want to model a compressible granular material, which is relevant for snow avalanches and other geophysical flows, we set the particles’ Young’s modulus to $E=10^{5}\,$Pa. This Young’s modulus *E* allows for substantial overlap of particles in the simulation if they are subject to a compressive load and thus mimics the material’s compressibility. We discuss our approach to model soft particles with a low particles’ Young’s modulus *E* in Sect. 4.4 and further analyze how varying *E* influences the results of our study in the Supplementary Material S.2 .

In DEM the material behavior is governed not only by the particle properties but also by the contact model, which is applied whenever particles interact. To simulate a cohesive granular material we use the *parallel-bond* model, originally developed for rock modeling [22]. This contact model has also proven to be suitable to simulate other cohesive geomaterials, such as sand [23], debris [24] and snow [20, 25–27].

The contact model consists of a linear and a cohesive component in parallel. The linear viscoelastic component consists of a spring and a dashpot in the normal direction and a spring and a coulomb friction limit in the tangential direction. The cohesive component is a bond connecting the particles in parallel to the linear component. The bond acts mechanically like a beam and can sustain tensile, bending, shear and torsional forces. In our simulations a new bond is formed whenever two unbonded particles make contact.

In order to assess the influence of cohesion in the pressure build-up processes, we perform simulations of a cohesive and a cohesionless scenario. In the cohesionless and the cohesive case we implement a cohesive strength of $\sigma _{coh}=0.0$ kN/m$^2$ and $\sigma _{coh}=10.0$ kN/m$^2$, respectively. This value corresponds to the tensile and pure shear strength of the cohesive bond.

**Table 1:** Granular material properties (a) and simulation setup parameters (b)

Parameter	Symbol	Unit	Value
*a) Particle and contact properties*			
Particle density	$\rho _{p}$	kg/m$^{3}$	500
Particle diameter	$d_{p}$	m	$0.08\pm {}0.008$
Young’s modulus	*E*	Pa	$10^{5}$
Friction coefficient	$\mu$	−	0.5
Restitution coefficient	$e_{r}$	−	0.05
Cohesive strength	$\sigma _{coh}$	N/m$^2$	$0.0, 10^{4}$
*b) Simulation setup parameters*			
Domain length	$D_x$	m	11, 22
Domain width	$D_y$	m	28
Domain height	$D_z$	m	28
Flow velocity	*v*	m/s	3
Flow height	*h*	m	2.5
Obstacle width	*w*	m	$0.24-6.0$

### Compression tests of the granular material

One of this study’s main objectives is to physically link the properties of the MD to the impact pressure on the obstacle. Hence, we need to bridge the gap between the relevant processes at the micro scale of the particles, such as the force chains and particle densification [12, 14], and the forces on the obstacle at the macroscopic scale in which we are interested. To achieve this, in this section we characterize the behavior of the granular material presented in the previous section under compressive loading by performing displacement-controlled axial compression tests.

**Fig. 3:**
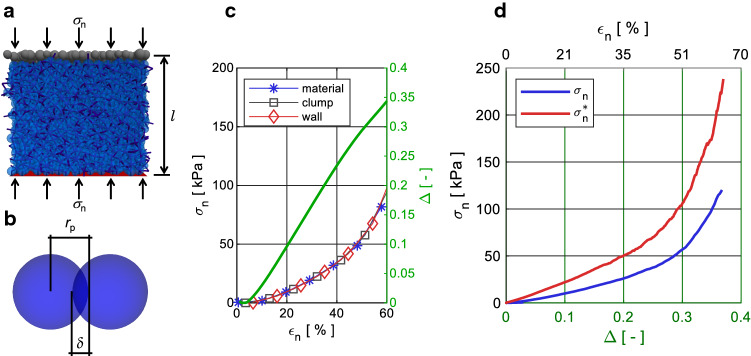
Panel a shows the setup of the compression test, where the material (blue particles and contact network) is compressed between a wall (red) at the bottom and a clump (gray) at the top. For better visibility the particles are not to scale with the sample size. Panel b shows two spherical particles with radius $r_p$ and interpenetration $\delta$. Panel c shows a comparison between $\sigma _n$ measured at the wall (red), at the clump (gray) and inside the granular material (blue), as well as the resulting $\varDelta$ (green, right y-axis) in the cohesionless case as a function of the strain $\epsilon _n$. Panel d shows the dependency between the normal stress $\sigma _{n}$ in the granular material and the relative interpenetration $\varDelta$ (bottom x-axis), as well as the macroscopic strain $\epsilon _{n}$ (top x-axis) for the cohesionless ($\sigma _{coh}=0.0$ kN/m$^2$, blue) and the cohesive ($\sigma _{coh}=10.0$ kN/m$^2$, red) case (color figure online)

We visualize the setup of the compression tests in Fig. 3a. For the compression test we use a material sample with a rectangular cross-section of equal side length of $s_0=6.0$ m, which corresponds to the maximum considered obstacle width *w*. A sensitivity analysis (Supplementary Material S.3) on the sample size shows that the compression tests’ results converge towards the results obtained with $s_0=6.0$ m.

In the normal direction the granular material is compressed between a wall and a collection of rigidly connected particles, referred to as a *clump*. In the lateral directions the tested material sample is confined by periodic boundaries. We perform the compression tests in a zero gravity environment.

On the macroscopic scale the compressive strain $\epsilon _n=(l_0-l)/l_0$ evokes a stress $\sigma _{n}$ in the compression direction on the boundary wall, on the boundary clump and inside the granular material.

At the micro scale the rigid particles in contact typically interpenetrate each other due to compressive loading. The particle interpenetration $\delta$ visualized in Fig. 3b evokes a force at the contact according to the contact law. In the following we express the compression of the granular material at the micro scale as the particle interpenetration normalized by the particle radius $\varDelta =\delta /r_p$.

Figure 3c shows the normal stress $\sigma _n$ inside the granular material, on the clump and on the wall as a function of the particle interpenetration in the cohesionless case. Figure 3d shows a comparison of $\sigma _n$ as a function of $\varDelta$ in the cohesionless and the cohesive case. To distinguish between the test with the cohesive and cohesionless granular material, we use an asterisk for the quantities in the cohesive scenario, e.g. $\sigma ^*_n$.

Panel c in Fig. 3 shows that the normal stress inside the granular material increases monotonically with increasing compression of the granular material. The concave shape of the $\epsilon _n$-$\sigma _{n}$ curve indicates that the normal stress $\sigma _{n}$ in the material increases at a higher rate than the interpenetration $\varDelta$ for large compressive strains. The stresses inside the granular material, on the wall and on the clump are almost identical. Hence, in the following sections we always refer to the normal stress $\sigma _n$ inside the granular material, which also acts on an obstacle wall in the case where there is one.

Panel d in Fig. 3 reveals that the cohesive granular material transmits a $\sim {}1.5$ times greater normal stress $\sigma ^*_{n}$ than the cohesionless material $\sigma _{n}$ for the same interpenetration $\varDelta$. We use the results of these compression tests to relate the particle interpenetration $\varDelta$ to the internal stress $\sigma _n$ in the material. In the following sections we use the notation $\sigma _{n}\,(\varDelta )$ when relating the two quantities on the basis of the graphs in Fig. 3.

### Definition of the mobilized domain

When a flow interacts with an obstacle we can generally distinguish between two domains. One, at a distance to the obstacle where the flow is not influenced, and another, in the vicinity of the obstacle where the flow is affected by the presence of the obstacle [28]. For the latter we use the term *mobilized domain* (MD).

We systematically identify the MD by analyzing the normal contact forces $F_n$ between the particles. Because the contacts between the particles are at random locations in the flow field, we discretize the flow domain with a regular grid to obtain a definition of the MD, which is consistent for all simulations. We use the average of the normal contact forces $F_n$ located inside the grid cell as the representative value for the whole cell. A grid cell typically contains more than 15 contacts and is 0.1 m, 0.1 m and 0.3 m in size in the *x*, *y* and *z* direction, respectively.

We choose the normal component of the contact force because the impact pressure on the obstacle physically originates from the force chains [7]. Thus, we consider the normal component to be the most relevant for the transmission of the pressure to the obstacle. Indeed, the analysis can also be performed using the shear component, leading to similar results [29]. Finally, we obtain the threshold value by defining a fixed percentile value of the averaged normal contact forces $F_n$ in the discretized grid. More in-depth details of how we define the threshold value are provided in Supplementary Material S.4.

Once the threshold value is obtained, we can define flow regions. The region where $F_n$ is greater than the threshold is considered to be within the MD. Anywhere that $F_n$ is lower is outside of the MD. Therefore, for very low percentile values the whole flow domain is considered the MD, while for very high percentile values the MD vanishes altogether. Hence, a physically relevant threshold value must be in between the extreme values.

For the present study we choose the 80th percentile of the normal contact forces as the threshold value. A sensitivity analysis is provided in Supplementary Material S.5 of this article. The analysis reveals that our results only weakly depend on the choice of the threshold in the range of the 70th to 90th percentile. Moreover, in Supplementary Material S.5 we visualize the change in MD for a sample simulation for the 70th, 80th and 90th percentile threshold values.

**Fig. 4:**
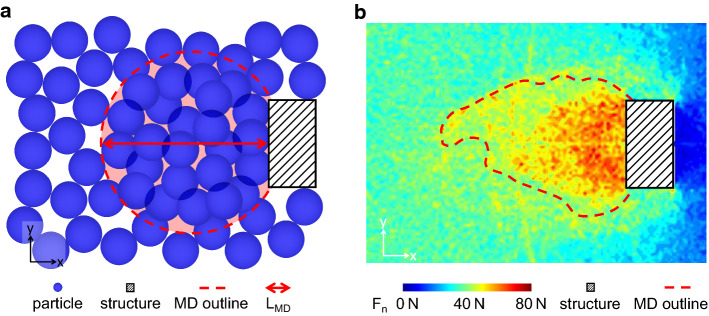
Panel a shows a schematic representation of particles (blue) interacting with an obstacle (hatched black, not to scale) and the MD (red dashed outline and shading). The red arrow shows the length $L_{MD}$ of the MD extent in the streamwise direction. Panel b shows the MD (red dashed outline) extracted from a simulation of an obstacle with a rectangular cross-section of $w=3$ m. The coloring of the field scales with the normal contact force $F_n$, which we use to define the MD (color figure online)

In Fig. 4 we show a horizontal section through the flow visualizing the definition of the MD schematically (panel a) and for the example of a 3 m-wide rectangular obstacle (panel b). For our analysis we use four quantities of the MD: (1) the mean streamwise velocity $v_{MD}$ inside the MD, (2) the volume $V_{MD}$ of the MD, (3) the length $L_{MD}$ of the MD extent in the streamwise direction, and (4) the averaged interpenetration $\varDelta _{MD}$ of the particles inside the MD.

As mentioned in Sect. 2.1, we only analyze MD properties at the last time step of the simulation. This is necessary because the vast number of particles and contacts in the system lead to large amounts of data, which cannot be stored for many time steps for all simulations. In Figs. 8 and 9, where we link the impact pressure to the MD properties, we consistently report the impact pressure value of the last time step of the simulation.

## Results

In the following section we first show how the impact pressure depends on the obstacle width and geometry, as well as on the cohesion of the granular material. In the same section we also compare the extent and the properties of the MD in cohesionless flows for different obstacle geometries. Thereafter, we show how cohesion affects the granular material in the MD and how this is linked to the change in impact pressure. Finally, we estimate the impact pressure exerted by the cohesionless flow on the obstacles based on the physical properties of the MD and compare the result with the simulated impact pressure.

### Influence of the obstacle width and geometry on the impact pressure and the MD

**Fig. 5:**
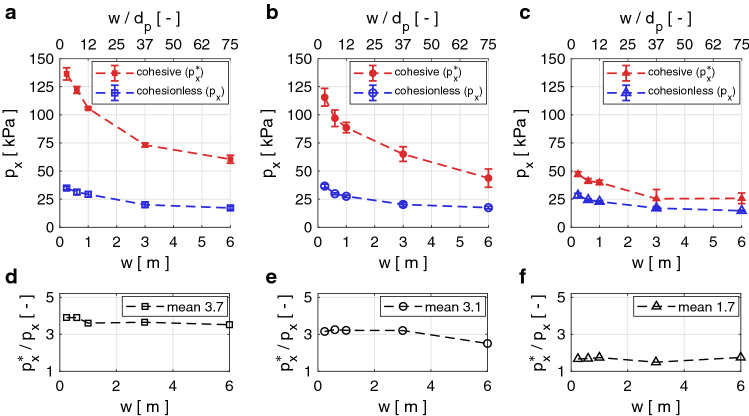
Panels  **a**, **b** and **c** show the impact pressure on obstacles of varying widths *w* with rectangular, circular and triangular cross-sections, respectively. The blue and red curves show the impact pressure exerted by a cohesionless and a cohesive flow, respectively. The top x-axis shows the width of the obstacle relative to the particle diameter $w/d_p$. The error bars indicate the standard deviation from the mean value of the pressure. Panels  **d**, **e** and **f** show the impact pressure ratio of cohesive and cohesionless flows on obstacles of varying widths *w* with rectangular, circular and triangular cross-sections, respectively

As described in Sect. 2.2, we study the pressure $p_x$ contributing to the impact force in the flow direction. Fig. 5 shows $p_x$ as a function of the obstacle width *w* for the obstacles with a rectangular, circular or triangular cross-section. On the top *x*-axis we indicate the ratio of obstacle width *w* to particle diameter $d_p$. This ratio may be critical for the interaction processes when the size of the particle is of the same order as the width of the obstacle [1, 30].

In Fig. 5, we observe that the impact pressure $p_x$ on all geometries decreases in a non-linear fashion for increasing obstacle width *w*. The impact pressure is highest on the obstacles with the rectangular and circular cross-sections, while it is significantly lower on the triangular obstacle. In the cohesionless cases the maximum pressure on the rectangular and cylindrical obstacles is $\sim {}1.3$ times higher than the pressure on the triangular obstacle. The average impact pressure exerted by the $v=3$ m/s and $h=2.5$ m cohesionless flow on all considered obstacle geometries lies between 15 kPa and 37 kPa. In the cohesive case the pressures on the narrowest $w=0.24$ m and the widest $w=6$ m obstacle vary considerably: $61-137$ kPa, $44-116$ kPa and $26-47$ kPa for the rectangular, circular and triangular cross-sections, respectively. Hence, as visualized in Fig. 5d–f for our cohesive scenario where $\sigma _{coh}=10.0$ kN/m$^2$, the impact pressure is approximately 3.7, 3.1 and 1.7 times higher than in the cohesionless case for the rectangular, circular and triangular cross-sections, respectively. Similarly, the maximum pressure on the rectangular and cylindrical obstacles is $\sim {}2.5$ times higher than the pressure on the triangular obstacle, which is higher than the pressure differences between the different geometries in the cohesionless case. It is important to note that these are approximate average values which vary for the different widths, as Fig. 5 clearly shows.

**Fig. 6:**
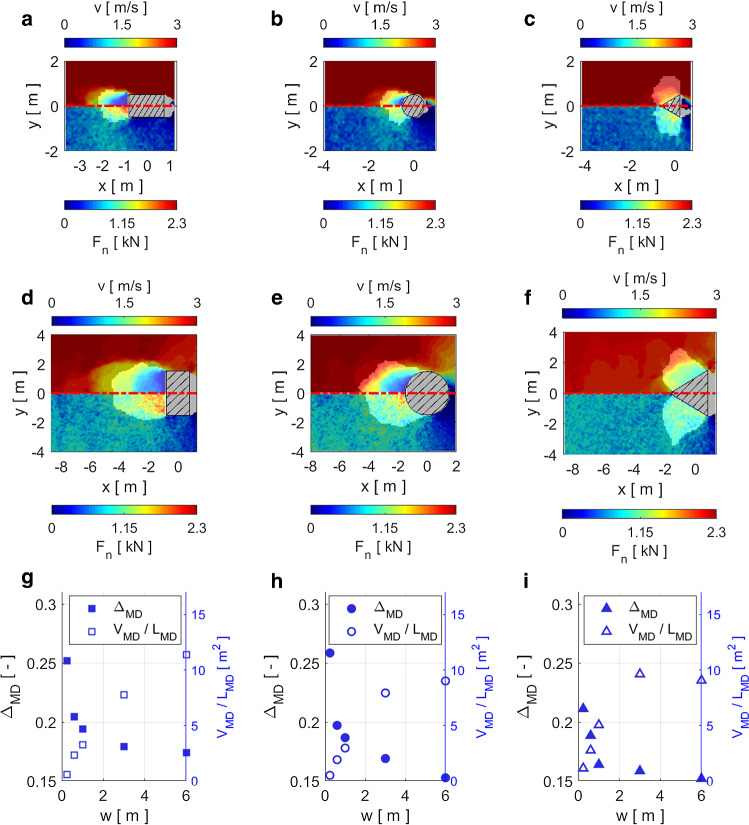
Analysis of the velocity field, the contact forces and the MD in cohesionless flows around the obstacle. The left, middle and right columns show the results for the rectangular, circular and triangular cross-sections (hatched areas), respectively. Panels **a**–**c** and **d**–**f** show obstacles with width $w=1$ m and $w=3$ m, respectively. Panels **a**–**f** show the velocity field in the upper half and the contact forces in the lower half. The region outside the MD is shadowed with a semi-transparent overlay. Panels **g**–**i** show the physical properties $\varDelta _{MD}$ (filled symbols, left y-axis) and $V_{MD}/L_{MD}$ (open symbols, right y-axis) of the MD for different obstacle widths *w* (color figure online)

In Fig. 6a–f we visualize the mobilized domains in the cohesionless case for all geometries by shadowing the region outside the MD with a semi-transparent overlay. The white area trailing the black and white hatched obstacle cross-section is a particle-free region caused by the detachment of the flow from the obstacle contour. The colored plots show the velocity field (upper half of plots) and the contact forces (lower half) in the vicinity of the obstacle in a horizontal section at mid-flow depth. Panels a–f therefore show that the MD has a distinct shape for all three geometries. The size of the MD scales approximately proportionately to the width of the obstacle. Moreover, in Fig. 6a–f we demonstrate that the extent of the zone influenced by the obstacle is mostly consistent between the contact forces and the velocity field.

In the panels g–i of Fig. 6, we show how the physical parameters $\varDelta _{MD}$ and $V_{MD}/L_{MD}$ of the MD depend on the obstacle width *w*. We use these quantities in Sect. 3.3 to estimate the impact pressure on the obstacle in the cohesionless case.

Figure 6g–i shows that the average particle interpenetration $\varDelta _{MD}$ inside the MD decreases with increasing obstacle width *w*, similarly to the impact pressure in Fig. 5. Because $\varDelta _{MD}$ reflects the interpenetration of the particles at the micro scale, we use it as an indicator of the compression of the material inside the MD. $\varDelta _{MD}$ ranges from 0.15 to 0.26 for the rectangular and cylindrical obstacles and from 0.15 to 0.21 for the triangular obstacle. Hence, similar to $p_x$, $\varDelta _{MD}$ is mostly highest for the rectangular obstacles, followed by the cylindrical obstacles, and is lowest for the triangular obstacles.

The volume-to-length ratio $V_{MD}/L_{MD}$ is a measure of the size of the MD and how far upstream the MD extends from the obstacle. $V_{MD}/L_{MD}$ increases almost linearly with obstacle width and levels off slightly for the obstacles with $w\ge {}3$ m. For the 6 m-wide cylindrical and triangular obstacles $V_{MD}/L_{MD}$ is considerably lower than the linear trend. The deviation from the linear trend may be a consequence of the MD occupying a large portion of the simulation domain for the widest obstacles with $w=6$ m.

**Fig. 7:**
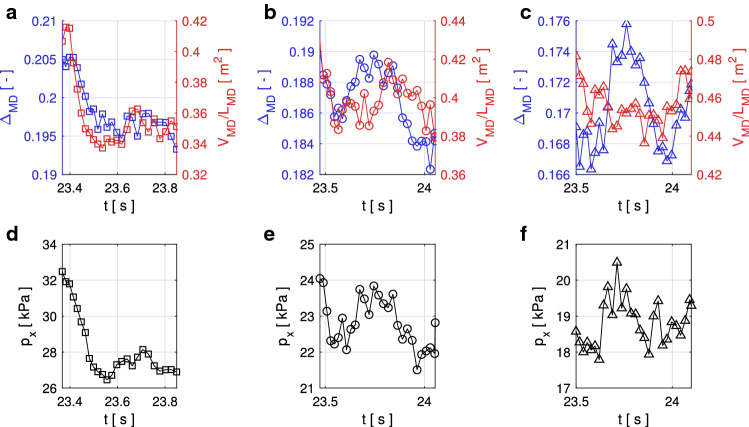
Comparison between time series of $V_{MD}/L_{MD}$ (red), $\varDelta _{MD}$ (blue) (panels **a**–**c**) and $p_x$ (panels **d**–**f**). The first, second and third columns show the data for the obstacles with rectangular, circular and triangular cross-sections, respectively (color figure online)

The question arises as to whether the simulated MD, as well as the link between the MD properties and the pressure in the last time step is representative of the temporal evolution of these quantities during the simulated time. To that end, we compare the temporal evolution of $V_{MD}/L_{MD}$ and $\varDelta _{MD}$ with $p_x$ in Fig. 7. Figure 7 shows the data for the examples of obstacles with $w=1$ m impacted by cohesionless flows. For these plots we select the same time window as used to average the impact pressure for the data shown in Fig. 5.

Although all quantities shown in Fig. 7 exhibit temporal fluctuations, there is a striking similarity between the qualitative temporal evolution of $\varDelta _{MD}$ (blue curves) in the upper plots and the black curves for $p_x$ in the lower plots. $V_{MD}/L_{MD}$, which we interpret as a measure of the spatial extent of the MD, also shows mostly good agreement with $p_x$, except in Fig. 7c and f. There the qualitative behavior of $\varDelta _{MD}$ and $V_{MD}/L_{MD}$ are almost inverted, indicating—in agreement with Fig. 6g–i—that $V_{MD}/L_{MD}$ probably plays a subordinate role in the impact pressure compared with $\varDelta _{MD}$.

### Influence of cohesion on the impact pressure and the MD

In this section we investigate the difference between the MD in the cohesionless ($\sigma _{coh}=0.0$ kN/m$^2$) and the cohesive ($\sigma _{coh}=10.0$ kN/m$^2$) case in order to understand the pressure difference observed between the two cases. In order to investigate the origin of the impact pressure increase due to cohesion, here we consider $\varDelta _{MD}$ to reflect the material compression inside the MD.

**Fig. 8:**
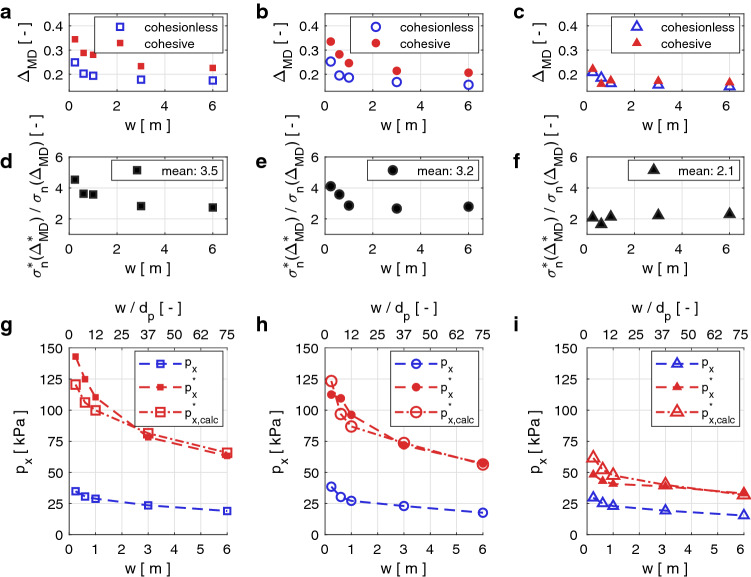
Influence of cohesion on the contact network inside the MD. The open blue symbols and red filled symbols represent data from the cohesionless and cohesive cases, respectively. Data for the obstacles with rectangular, circular and triangular cross-sections are shown in the left, middle and right columns, respectively. All panels show how the respective quantities vary with the obstacle width *w*. Panels **a**–**c** show the relative particle interpenetrations $\varDelta$. Panels **d**–**f** show the ratio of the normal stresses in the cohesive and cohesionless case $\sigma ^*_{n}(\varDelta ^*_{MD})/\sigma _{n}(\varDelta _{MD})$. Panels g–i show a comparison between the simulated impact pressure exerted by the cohesive flow and the pressure estimation calculated from the impact pressure in the cohesionless case multiplied by the normal stress ratio $p^*_{x,calc}=p_x\,\sigma ^*_{n}(\varDelta ^*_{MD})/\sigma _{n}(\varDelta _{MD})$ (open red symbols) (color figure online)

Figure 8a–c shows that the relative interpenetrations $\varDelta _{MD}$ in the cohesive case decrease for obstacles of increasing width, similarly to the cohesionless case already shown in Fig. 6g–i. The difference in $\varDelta _{MD}$ between the cohesionless and the cohesive case is $\sim {}4$ times larger for the rectangular and cylindrical obstacles than for the triangular obstacles and is larger for narrow obstacles $w\le {}1$ m than for wider obstacles.

In order to estimate how much more stress is transmitted by the cohesive granular material compared with the cohesionless flow, we convert the interpenetration in the MD to normal stresses $\sigma ^*_{n}(\varDelta ^*_{MD})$ and $\sigma _{n}(\varDelta _{MD})$ based on the results of the compression tests in Fig. 3d. The panels d–f in Fig. 8 show the ratio of the normal stresses $\sigma ^*_{n}(\varDelta ^*_{MD})/\sigma _{n}(\varDelta _{MD})$. This ratio reflects the stress level inside the MD in the cohesive material relative to the cohesionless case. While $\sigma ^*_{n}(\varDelta ^*_{MD})/\sigma _{n}(\varDelta _{MD})$ shows a decreasing tendency for obstacles of increasing width, the mean values are 3.5, 3.2 and 2.1 for the rectangular, circular and triangular geometries, respectively.

To test how the relative change in the normal stress in the MD between the cohesionless and the cohesive case is related to the difference in impact pressure, we multiply the pressure of the cohesionless case $p_x$ by the normal stress ratio $\sigma ^*_{n}(\varDelta ^*_{MD})/\sigma _{n}(\varDelta _{MD})$. Hence, we calculate the estimated impact pressure $p^*_{x,calc}$ of the cohesive scenario according to equation (1).

1 $$\begin{aligned} p^*_{x,calc}=p_{x}\,\sigma ^*_{n}(\varDelta ^*_{MD})/\sigma _{n}(\varDelta _{MD}) \end{aligned}$$

Figure 8g–i shows the estimated impact pressures $p^*_{x,calc}$ for all obstacle geometries. From these panels we observe that the stress ratios $\sigma ^*_{n}(\varDelta ^*_{MD})/\sigma _{n}(\varDelta _{MD})$ agree well with the impact pressure increase due to cohesion.

### Analytical model to quantitatively link the MD properties to the impact pressure

In this section we use the principle of force balance to estimate the impact pressure exerted by cohesionless flows on obstacles. To that end, the simulation outcomes (e.g., the MD size) are used as inputs to the analytical model. Note that for this reason, this model has no predictive capacity. The purpose is to highlight the physical link between the properties of the MD and the impact pressure.

Similarly to in other studies [16, 17], we divide the impact force into a kinetic $F_{x,k}$ and a gravitational $F_{x,g}$ contribution. Hence, the calculated pressure $p_x$ is the sum of the two contributions divided by the frontal area $A_{yz}$:

2 $$\begin{aligned} p_{x,calc}=(F_{x,k}+F_{x,g})/A_{yz}=p_{x,k}+p_{x,g} \end{aligned}$$

The individual contributions are calculated as follows:

1. Kinetic contribution:
  3 $$\begin{aligned} F_{x,k}=\frac{1}{2}\,(v^2-v_{MD}^2)\frac{m_{MD}}{L_{MD}} =\frac{\rho }{2}\,(v^2-v_{MD}^2)\frac{V_{MD}}{L_{MD}} \end{aligned}$$
  We calculate the kinetic contribution based on the change in kinetic energy of the granular mass $m_{MD}=\rho { }V_{MD}$, initially travelling at the free flow velocity *v*, which is decelerated to the mean velocity of the particles in the MD $v_{MD}$ within the streamwise extent $L_{MD}$ of the MD, due to the obstacle’s resistance to the flow.
2. Gravitational contribution:
  4 $$\begin{aligned} F_{x,g}=\zeta {} \frac{1}{2} \rho g h^2 w \end{aligned}$$
  The gravitational contribution is calculated as a hydrostatic-like force increasing with the flow depth squared [7]. The factor $\zeta =\sigma _{n} (\varDelta _{MD})/\sigma _{z}$ reflects the stress concentration inside the MD due to the densification of the contact network $\sigma _{n} (\varDelta _{MD})$ with respect to the hydrostatic stress $\sigma _z=\rho g h$.

**Fig. 9:**
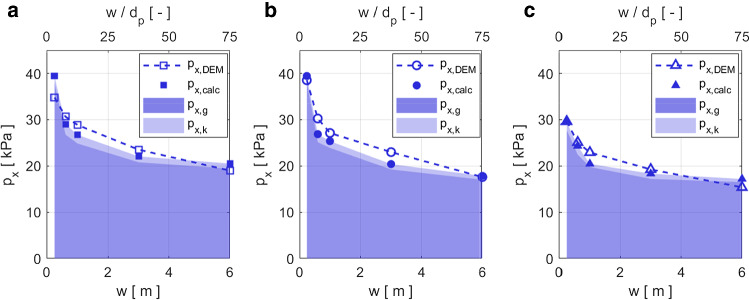
The blue symbols in panels **a**, **b** and **c** show the comparison of the calculated impact pressure according to equations (2) to (4) with the simulated impact pressure of a cohesionless flow on obstacles of varying widths *w* with a rectangular, circular and triangular cross-sections, respectively. The top x-axis shows the width of the obstacle relative to the particle diameter $w/d_p$. The dark and light blue shaded areas represent the calculated gravitational and kinetic contributions, respectively (color figure online)

Figure 9a–c shows a comparison of the impact pressures calculated with equations (3) to (4) and the simulated pressures in the cohesionless scenario for all obstacle geometries and widths. The shaded areas represent the calculated contributions $p_{x,g}$ (dark shade of blue) and $p_{x,k}$ (light shade of blue), respectively. The calculated pressure is the sum of the gravitational and the kinetic contribution and is shown by the filled blue symbols.

The calculated impact pressure $p_{x,calc}$ shown in Fig. 9 decreases non-linearly for obstacles of increasing width. Hence, it agrees well with the qualitative trend of the simulated impact pressure $p_{x,DEM}$. For all cross-sections the calculation overestimates the simulated impact pressure for the narrowest ($w=0.24$ m) and widest obstacles ($w=6$ m). The impact pressure on the obstacles of intermediate width (0.6 m$\,\le {}w\le {}3$ m) is slightly underestimated. For the rectangular and the cylindrical obstacles $p_{x,calc}$ is lower than $p_{x,DEM}$ for the narrow obstacles ($w\le {}1$ m), while it is larger for the wide obstacles ($w\ge {}3$ m). The average relative error between the calculated and the simulated impact pressure is $7\,\%$ for all cross-sections.

For all calculations $p_{x,g}$ contributes $95\,\%$ and $p_{x,k}$ 5 % to the total impact pressure $p_{x,calc}$. This highlights that, for the present case where $Fr=0.61$, both contributions are present and the gravitational contribution is considerably larger than the kinetic contribution. While $p_{x,k}$ is almost constant for all widths and geometries, $p_{x,g}$ varies similarly to the simulated impact pressure $p_{x,DEM}$.

## Discussion

### Physical processes governing the flow–obstacle interaction and impact pressure for varying obstacle geometries

The simulated impact pressure shown in sSect. 3.1 is in agreement with previous research showing that the pressure on obstacles offering high resistance to the flow is higher than for pointed obstacles [18, 31]. In [20], we performed a study on the avalanche pressure on a particular geometry of an existing instrumented steel pylon [32], and we concluded that the size of the MD and the resulting impact pressure build-up depends on the obstacle geometry. The evident variation in MD size in Fig. 6 and the impact pressure in Fig. 5 for the differing obstacle geometries and widths confirms this dependency. The linearly increasing trend of the MD size parameter $V_{MD}/L_{MD}$ for obstacles with increasing width for $w<6$ m is consistent with the result in Fig. 8a in [16]. Faug [16] reports that a linear increase of the MD’s typical length scale is a robust feature appearing in a number of experimental and numerical studies on the impact of granular flows on obstacles.

Interestingly, we find that the average pressure $p_x$ on the obstacle decreases non-linearly for obstacles of increasing width *w*. This phenomenon is known to occur with creeping snow and granular snow avalanches [28, 33]. Studies on the relative motion of intruders in dense granular materials at low *Fr* often report the drag force on the intruder. In these cases the drag force increases with the size of the intruder [10, 30, 34]. However, if the data from these studies is analyzed with respect to the pressure rather than the force, the trend of decreasing impact pressure for increasing intruder size is confirmed (Supplementary Material S.6).

A tentative explanation for the decreasing pressure on obstacles of increasing width can be given for the obstacles with rectangular cross-sections. Figure 4 b demonstrates shows that an arch of strong contact forces forms upstream of the edge facing the flow, especially for wide obstacles $w\gtrsim {}1$ m with rectangular cross-sections. Hence, the middle part of the obstacle is largely sheltered from the impact of the incoming flow by the arch. However, this fails to explain the decrease in impact pressure on the circular and triangular cross-sections of increasing width, because the formation of the arch is not evident, e.g. in Fig. 6e–f.

More comprehensively, for varying obstacle widths of all cross-sections, the particle interpenetrations in MD (Fig. 6g–i) and the impact pressure (Fig. 5) qualitatively show a high degree of similarity. This suggests that the impact pressure is governed by the compression state of the granular material inside the MD. In the MD of the narrow obstacles the particles are jammed closer together than in the MD of the wider obstacles. From the compression test in Fig. 3d we learn that increasing $\varDelta _{MD}$ leads to an increased stress level in the granular material, resulting in a higher pressure on the obstacle. In the absence of rigorous evidence, we speculate that $\varDelta _{MD}$ is lower for wide obstacles because of the higher shearing of the material, which is necessary for the particles to travel around the obstacle. This causes higher shear dilation and leads to a looser packing of the particles in the MD of wide obstacles. This is consistent with the finding of Seguin et al. [29], who show that although the material inside the MD is almost stagnant, zones of high shear and dilation are located in the vicinity of the upstream boundary of the obstacle.

As shown in Fig. 6g–i, we identify the varying degrees of material compression in the MD $\varDelta _{MD}$ as the predominant origin of the differing impact pressure on the three geometries. Obstacles offering high resistance to the flow, such as the rectangular and circular cross-section in Fig. 6g and h, cause the granular material to jam upstream, which leads to high $\varDelta _{MD}$. Pointed obstacles, such as the triangular cross-section in Fig. 6i, tend to deflect the flowing material without causing particle jamming. This leads to lower $\varDelta _{MD}$ and consequentially to lower impact pressure.

Hence, in our results we can consistently correlate $\varDelta _{MD}$ to the impact pressure on obstacles of varying geometry and width. This provides further evidence supporting the assumption of Chehata et al. [8] that the “granular drag must result from the compressive stresses acting on the upstream stagnation region”.

As shown in Figs. 6, 8 and 9, we are able to show that the instantaneous impact pressure and MD properties at the last simulation time step correlate for various obstacle geometries, for cohesive and cohesionless flows. Figure 7 confirms for three example simulations that the instantaneous impact pressure and MD properties also correlate for most points in time in the simulated flow. Hence, as the flow characteristics of real avalanches and other gravity-driven granular flows evolve over time, we are confident that the link between the instantaneous MD properties and impact pressure is still valid for subcritical granular dense flows interacting with obstacles. When considering applications to structural engineering, note that real-world scenarios may deviate substantially from the steady subcritical granular dense flow considered here. Among other things initial impact or large material accumulations upstream of the obstacles may critically damage the obstacle.

### Influence of cohesion on the flow–obstacle interaction processes

The results displayed in Fig. 5 confirm previous research that a cohesive flowing granular material exerts significantly more pressure than the same material without cohesion [14, 20]. Indeed, in a previous study we found a scaling (see Supplementary Material S.7) for the pressure increase due to cohesion, relating the impact pressure increase to the ratio of *Fr* and the Bond number *Bo*, where *Bo* is the cohesive strength $\sigma _{coh}$ normalized by the vertical stress inside the granular material [20]. When evaluating the scaling for the Froude and Bond numbers in the present study, we calculate a pressure increase factor of $\sim {}2.3$. Considering that the impact pressure increase due to cohesion may vary as a result of differing obstacle geometries, this is in the range of impact pressure increase factors of 1.7–3.7, as observed in Fig. 5.

In Fig. 8a–c, we observe that in the cohesive case the particle interpenetration $\varDelta _{MD}$ in the MD is larger than in the cohesionless case. We assume that a higher $\varDelta _{MD}$ arises because the cohesive granular material sustains more loading from the upstream flow before rearrangements of the force chains within the granular material allow the particles to flow around the obstacle. On the particle scale the force chain rearrangement is inhibited by the cohesive bonds connecting the particles. Similarly to the increase in impact pressure (Fig. 5) due to cohesion, we also observe a greater increase in particle interpenetration $\varDelta _{MD}$ for the rectangular and cylindrical obstacles than for the triangular obstacle.

Using $\varDelta _{MD}$ and the compression tests (Fig. 3d), we calculate the ratio of the stress inside the granular material in the cohesive and cohesionless cases (Fig. 8d–f). Based on the analysis of the cohesionless flows (Sect. 4.1), we suspect that the pressure increase due to cohesion also originates from the jammed material state inside the MD. Hence, multiplying this ratio by the impact pressure of the cohesionless scenario according to equation (1) gives us an estimate of the impact pressure increase due to cohesion. A comparison between the simulated impact pressure $p^*_x$ and $p^*_{x,calc}$ in Fig. 8g–i shows that the factor $\sigma ^*_{n}(\varDelta ^*_{MD})/\sigma _{n}(\varDelta _{MD})$ mostly reproduces the pressure increase due to cohesion for most of the obstacles of differing widths and geometries. The small deviations between the simulated and the estimated cohesive impact pressure may be caused by secondary processes which do not scale proportionally with the width of the obstacle, such as the arch formation for the rectangular obstacles mentioned in Sect. 4.

Nevertheless, the good agreement between $p^*_x$ and $p^*_{x,calc}$ indicates that the pressure increase is a direct consequence of the enhanced stress transmission between cohesive particles compared with cohesionless particles.

The fact that $\sigma ^*_{n}(\varDelta ^*_{MD})/\sigma _{n}(\varDelta _{MD})$ varies for the different geometries highlights that the pressure increase due to cohesion depends not only on cohesion itself, but also on the obstacle geometry. We conclude that, the force transmission through the cohesive force chains is more efficient if the cohesion increases and, similar to the cohesionless case, if the flow impacts the obstacle surface at a right angle.

### Analytical model to quantitatively link the MD properties to the impact pressure

In order to establish a quantitative link between the MD and the impact pressure, we estimate the pressure based on the MD properties using an analytical model. Because estimating the MD properties is no less complex than determining the impact pressure itself, the model is descriptive rather than predictive. We model the impact pressure in the cohesionless case as the sum of a kinetic and a gravitational contribution, as suggested by previous studies [16, 17]. The results of Sect. 3.3 show that the pressure estimated with the two contributions (equations (2) to (4)) mostly reproduces the simulated impact pressure of the cohesionless scenario. The average relative error of the calculated pressure is $7\,\%$ compared with the simulated pressure $p_{x,DEM}$. Hence, the qualitative agreement of the calculated and the simulated impact pressure highlights again that the geometrical properties of the MD and the physical material properties within the domain govern the pressure on the obstacle. Furthermore, the agreement also indicates that a model considering a kinetic $F_{x,k}$ and a gravitational $F_{x,g}$ contribution is able to capture the main physical processes involved in the flow–obstacle interaction.

In literature the kinetic contribution $F_{x,k}$ of the drag force is often calculated using the empirical drag coefficient $C_D$. Previous studies suggest that $C_D$ can be divided into a flow regime and a part that is dependent on structure geometry [18, 35, 36]. In equation (3) it is the material’s deceleration $v^2-v_{MD}^2$ that accounts for the influence of geometry. For example, due to its shape with the wedge facing the flow, the flowing material is more deflected than decelerated in the case of the triangular obstacle compared with e.g. the rectangular cross-sections. This leads to a smaller velocity difference $v^2-v_{MD}^2$ for the triangular cross-section. This trend is analogous to the $C_D$ values usually reported in the literature, which are higher for the rectangular cross-section than for the triangular cross-section [37, 38].

The ratio of the MD volume and length, $V_{MD}/L_{MD}$, has the dimensions of an area and accounts for the size and the shape of the MD. In [20] we show that the shape and size of the MD is mainly controlled by *Fr*. Hence, we interpret the factor $V_{MD}/L_{MD}$ as the part of the drag coefficient that is dependent on the flow regime.

For the gravitational $F_{x,g}$ contribution, in their seminal article Albert et al. [7] introduce a proportionality factor that accounts for the particle properties. In equation (4) we define an analogous factor $\zeta$. In our formulation, however, the factor is defined as the ratio of the normal contact stresses derived from the particle interpenetration $\sigma _{n} (\varDelta _{MD})$ and the theoretical hydrostatic stress $\sigma _z$. $\zeta$ therefore considers the compressive behavior of the material inside the MD, which governs the impact pressure due to build-up of the MD.

In our flow scenario with $Fr=0.61$, we find that the calculated pressure is composed of 95 % gravitational and 5 % kinetic contributions. This agrees with the observation of Faug [16] that for a wide range of Froude numbers, $0.1\lesssim {}Fr\lesssim {}10$, both the kinetic and the gravitational contribution of the impact pressure are present. At low velocities the dominance of the gravitational pressure contribution is probably responsible for the fact that the pressure is often observed to be independent of the velocity in this range of low *Fr* [2, 7]. The kinetic contribution increases quadratically with increasing speed and consequentially outweighs the gravitational contribution only at higher velocities. When *Fr* becomes supercritical, the MD changes substantially from the rounded shapes in Fig. 6 to a bow shock [20, 39, 40]. Hence, it is not clear whether the interaction processes and the analytical model presented in this study still hold in the supercritical regime. Similar to what is observed in real experiments, the impact pressure contributions in DEM simulations cannot be identified individually. Simulations with higher flow velocities, where the kinetic contribution is dominant, could help to determine whether the kinetic contribution is adequately accounted for in our model.

Figure 9 shows that the calculated impact pressure $p_{x,calc}$ overestimates the simulated impact pressure of the obstacles with $w=0.24$ m and $w=6$ m, while the pressure on the obstacles of intermediate width 0.6 m $\le {}w\le {}3$ m is underestimated. This difference probably arises because of the varying proportion between the size of the whole simulation domain and the area occupied by the obstacle. Although we only consider a fraction of the domain for the MD threshold calculation, to keep the ratio between the considered domain size and varying obstacle sizes constant (Supplementary Material S.4), the error persists. The error could probably be reduced more efficiently by increasing the size of the simulation domain, which is currently not possible with the computational resources available.

### Limitations

Although we are able to find good agreement between the simulated and the estimated pressure using the MD properties, we identify three main limitations of the present analysis.

First, as described in Sect. 2.5, we use a percentile threshold of the normal contact forces to identify the MD, which is the basis for our results. We perform a sensitivity analysis (Supplementary Material S.4) and show that our results do not crucially depend on the threshold value within the range of the 70th to 90th percentile. Nevertheless, the deviations between the simulated and calculated impact pressure, as well as the deviation from the linear trend of $V_{MD}/L_{MD}$ for $w=6$ m in Fig. 6g–i show the limitations of our approach, as the MD identification is delicate. Hence, in the future it would be preferable to establish a threshold based on the physical properties of the material surrounding the MD to distinguish the MD from the rest of the flow domain. In order to achieve this, various variables can be considered potential candidates for identifying the MD or may be linked to the impact pressure, such as shear force, shear rate, bulk density, coordination number, velocity [8, 18], and stress anisotropy [41]. After testing a number of variables, we conclude that, among these variables, $\varDelta$ allows us to make the most comprehensive analysis.

Second, the computational resources needed for the DEM simulations are considerable. We cut computational costs by only simulating an isolated volume of granular material interacting with the obstacle for a relatively short period of a few seconds (Sect. 2.1). This implies that: (1) we neglect small perturbations in the far-field and only consider strong disturbances in the flow close to the obstacle, and (2) we work on mesoscopic timescales, which are longer than the time of readjustment of particle network, but potentially shorter than the time of evolution of the granular avalanche. The time scales of the granular flow vary because real geophysical flows vary in size and are highly non-stationary processes, where often the flow height, flow velocity, the material composition etc, changes before a steady-state is reached. Hence, while at the mesoscopic timescales of our simulations we can neglect acceleration terms in the force balance because the flow is almost in a steady state, this does not imply that the whole flow has reached a steady state. The simulations show that the MD of wide obstacles in cohesive flows takes longer to establish compared with the MD of narrow obstacles in cohesionless flows. Hence, in the case of narrow obstacles with $w\le {}1$ m, we are confident that a steady state impact force is reached within our simulations. For the interaction of wide obstacles and cohesive flows, even longer simulations are needed to confirm that the pressure does not change significantly anymore. Therefore, the pressure difference between the narrow and wide obstacles may be slightly overestimated here. However, the trend of decreasing average impact pressure on obstacles of increasing width has also been found in other studies (Supplementary Material S.6) and is therefore not an artefact of our numerical procedure. Moreover, in Sect. 3.3 we estimate the impact pressure based on the instantaneous MD properties and compare it to the impact pressure at the same time step. Hence, even when a steady state is not reached, the good agreement of the estimated and simulated pressure in the last simulation time step in Fig. 9 highlights once more how crucially the impact pressure is linked to the instantaneous physical properties of the MD.

Third, in the present analysis we simulate a granular material consisting of soft grains by using a low particle Young’s modulus *E*. Using a low *E* allows for large particle compression and thereby makes it possible to mimic the characteristics of snow aggregates in avalanches. The relevance of the model parameters and contact properties, such as the particle Young’s modulus *E*, have to be reviewed carefully when considering different granular materials or applications.

In snow avalanches the characteristics of the aggregates are assumed to depend on changing snow quality and interactions between aggregates leading to fracturing or further aggregation (e.g., [26, 42]). This may suggest that the Young’s modulus of the aggregates strongly depends on the snow type. However, in the absence of rigorous measurements of the aggregates’ mechanical properties in flowing avalanches, we chose a value of $E=10^5$ Pa, which is at the lower range of measured values for snow. Using this fixed value for *E*, we calibrated the other model parameters and successfully validated the model [20]. Moreover, in the Supplementary Material S.2, we present a detailed analysis of how varying *E* influences the results of our study. It is noted in particular that the choice of the elastic modulus has very little influence on the impact pressure, which further supports our choice.

## Conclusions

In this study, we demonstrate that the non-linear decrease in the impact pressure on obstacles of increasing width is linked to the compression of the granular material consisting of soft particles. Our results indicate that the particle interpenetration $\varDelta _{MD}$ inside the MD decreases in a similar fashion as the impact pressure on obstacles for increasing *w*. Furthermore, we show that the stress ratio of the cohesive to the cohesionless case $\sigma ^*_{n}(\varDelta _{MD})/\sigma _{n}(\varDelta _{MD})$ agrees well with the increase in the average impact pressure due to cohesion for different obstacle geometries. Hence, we identify two main mechanisms causing the pressure increase due to cohesion. First, the particle interpenetration $\varDelta _{MD}$ and thus the material compression in the MD is higher than in the cohesionless case, probably because the cohesive bonds inhibit the rearrangement of force chains and thus the particle flow around the obstacle. Second, the force transmission between the particles is higher than in the cohesionless case because the particles are connected more rigidly through the cohesive bond (Fig. 3). The analysis also shows that further processes might be present but play a subordinate role to the two processes mentioned above.

Finally, we estimate the impact pressure in the cohesionless case based on the MD properties and considering a kinetic and a gravitational contribution. The agreement of the calculated and the simulated impact pressure values provides a further indication that the MD fundamentally governs the pressure on the obstacle [8, 16].

By calculating the pressure of a cohesionless and cohesive granular flow impacting an obstacle using the physical properties of the MD, we show that the impact pressure exerted by a subcritical flow on an obstacle is quantitatively linked to the physical properties of the MD. The calculations highlight that the jamming and compression of the material inside the MD govern the pressure build-up on the obstacle.

We identify limitations of our model linked to the computational cost of the simulations and the MD identification. Nonetheless, in the future the presented method could help to estimate impact pressures on obstacles based on the jamming of the granular material predicted for specific geometries and using compression tests.

## Supplementary Information

Below is the link to the electronic supplementary material

Supplementary file 1 (PDF 821 kb).

## Data Availability

https://doi.org/10.5281/zenodo.4079357.

## Declarations

### Code availability

https://doi.org/10.5281/zenodo.4079357.
