# Supplementary material for: The concept of the mobilized domain: how it can explain and predict the forces exerted by a cohesive granular avalanche on an obstacle
Source: Granul Matter. 2022 Feb 11;24(2):45. doi: 10.1007/s10035-021-01196-1 (PMC8837560; doi:10.1007/s10035-021-01196-1)
Supplement: Supplementary file 1 — (PDF 821 kb) [file 10035_2021_1196_MOESM1_ESM.pdf]

Granular Matter
DOI: 10.1007/s10035-021-01196-1

Supplementary Material

for

The concept of the mobilized domain: how it can explain and predict the forces exerted by a cohesive granular avalanche on an obstacle

M. L. Kyburz[1,2,*], B. Sovilla[1], J. Gaume[1,3], and C. Ancey[2]

[1] WSL Institute for Snow and Avalanche Research SLF, Davos, Switzerland

[2] Environmental Hydraulics Laboratory, École Polytechnique Fédérale de Lausanne, Lausanne, Switzerland

[3] Snow and Avalanche Simulation Laboratory SLAB, École Polytechnique Fédérale de Lausanne, Lausanne, Switzerland

Received: 12 Nov. 2020 / Accepted: 1 Dec. 2021

This research was funded by the Swiss National Science Foundation in the framework of the project "Pressure on obstacles induced by granular snow avalanches" (grant no. 200021_169640).

* Corresponding author e-mail: kyburz@slf.ch

Content

This document contains supplementary material for the article “The concept of the mobilized domain: how it can explain and predict the forces exerted by a cohesive granular avalanche on an obstacle” and contains the following:
S.1: Temporal evolution of the impact pressure during simulations
S.2: Analysis on the influence of the particle Young’s modulus E on the results
S.3: Sensitivity analysis of axial compression tests
S.4: Definition of the mobilized domain (MD)
S.5: Sensitivity of the results on the MD threshold value
S.6: Impact pressure calculated from reported drag forces in the literature
S.7: Impact pressure scaling law for cohesive avalanches

S.1 **Temporal evolution of the impact pressure during simulations**

In section 2.1 of the main article we state that the reported impact pressure values are the temporal average of the pressure on the obstacle during the second simulation phase. Figure S.1 shows the temporal evolution of the impact pressure during simulations of a cohesionless flow (a–c) and a cohesive flow (d–f) for the examples with width $w = 1$ m for all cross-sections.

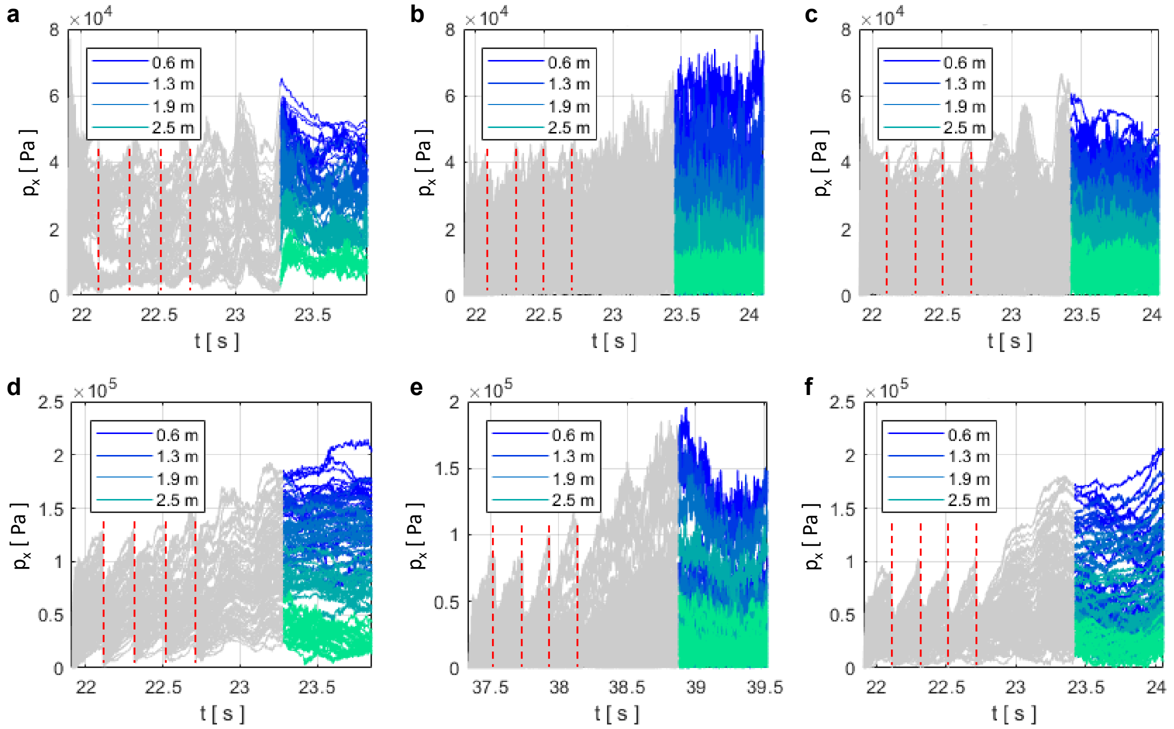

Fig. S.1 Time series of the simulated pressure in various positions on the obstacle (no distinction made) in the cohesionless (top row) and the cohesive (bottom row) scenario for an obstacle with a rectangular (left), circular (middle) or triangular (right) cross-section.

In the impact pressure graphs in Figure S.1 above, we can identify the fluctuations due to the generation of new particles in the first simulation phase (marked with red dashed lines).

The colored parts of the graphs in the second simulation phase (see main article section 2.1) are averaged to obtain the impact pressure values presented in the article. More specifically, we average only the last 30 % of the whole time series, including the first and the second phase, because we expect this time segment to be the closest to the steady state impact pressure.

S.2 Analysis on the influence of the particle Young's modulus E on the results

In this section we assess how our results depend on the particles' Young's Modulus E by performing compression tests (Fig. S.2) and granular flow–obstacle impact simulations (Fig. S.3) with materials of varying E.

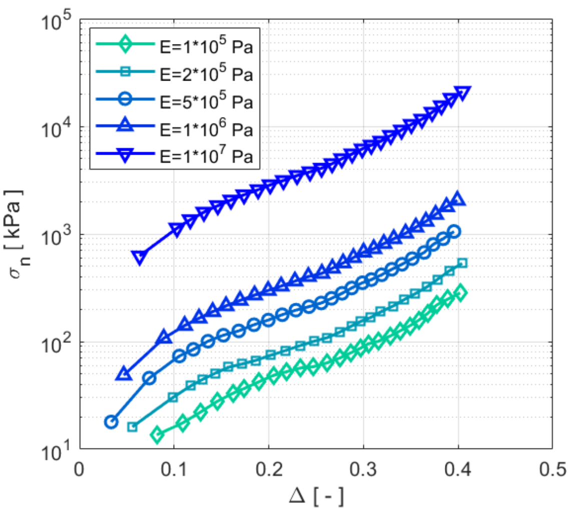

Fig. S.2 Dependency of the normal stress σ_n on the particle Young's modulus E in confined compression tests.

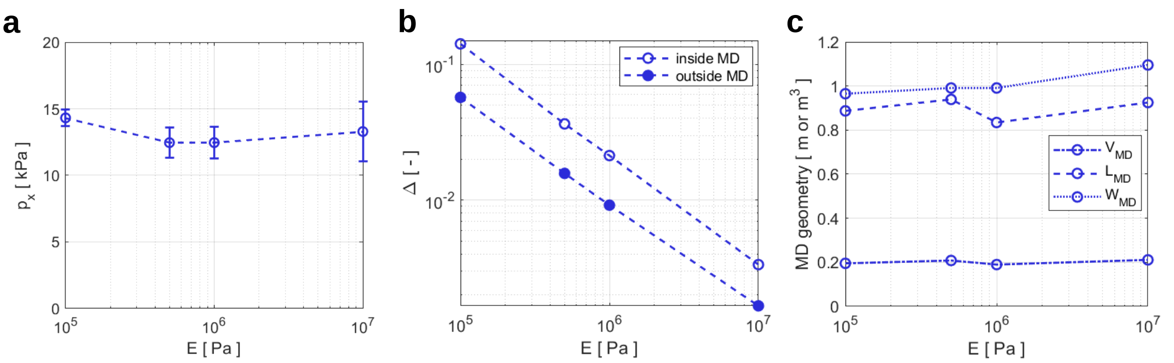

Fig. S.3 Panel a shows the average impact pressure p_x on a cylindrical obstacle with $w = 1$ m impacted by a plug flow of granular materials with varying E at 3 m/s. The error bars indicate the standard deviation from p_x averaged during simulation phase II (see section 2.1 in the main article). Panel b shows the relative particle overlap Δ in and outside of the mobilized domain for varying particle Young's modulus E. Panel c shows the dependency of streamwise length L_{MD}, transverse width W_{MD} and volume V_{MD} of the mobilized domain on the particle Young's modulus E.

Figure S.2 above shows that the axial stress σ_n in the compression tests increases with increasing E. The average impact pressure p_x in Figure S.3 a varies roughly 15 % with no clear trend when increasing particle Young's modulus E. The error bars in Figure S.3 a show that the pressure fluctuations increase with increasing E and that the pressure values for the simulations with $10^5 \leq E \leq 10^6$ Pa lay within the range of pressure fluctuations of the simulation with $E = 10^7$ Pa.
As expected, with increasing E the particle overlaps Δ decrease significantly as shown in panel b of Figure S.3. Panel c of Figure S.3 shows that the geometrical features of the mobilized domain (MD) are not strongly affected by the change in E. This robustness may be explained by the fact that we used a percentile criterion to identify the MD, which is not defined in absolute terms, but relative to the stresses in the material in the flow field.
We think that our results being dependent on E is neither an issue nor a contradiction: Here we simulated soft particles, as we want to model a compressible granular material, more specifically snow. The model parameters were calibrated and tested in Kyburz et al. [1] and confirmed in the present article by comparing the simulated impact pressure on an obstacle to measurements from the VdlS test site for various distinct avalanche scenarios. The compressibility of snow is well documented in the literature (e.g., [2,3,4,5]). Hence, in contrast to a large body of research, where models are calibrated to mimick rigid particles such as glass beads or sand, where volume change of the material should be avoided and the dynamics are governed by frictional inter-particle processes, here we specifically aim to model the compressive behavior of snow. We therefore use a particle Young's modulus of $E = 10^5$ Pa in all simulations, which is in the range of reported values from the literature [6,7,8,9].
Furthermore, to establish the link between the MD properties and the impact pressure we analysed the normal stress σ_n for a given particle compression Δ from compression tests and subsequently applied equations 2-4 (main article). Hence, if we change the contact model, e.g. such that the particles become rigid, the material response in the compression test changes as well. We wanted to test whether the link between MD properties and the impact pressure using our model still changed when E was altered. To that end, we performed compression tests and impact pressure simulations and applied the same methodology as in the article. Figure S.4 shows the comparison of simulated and estimated impact pressure in the last time step of the simulations similar to in Figure 9 in the manuscript for varying E.

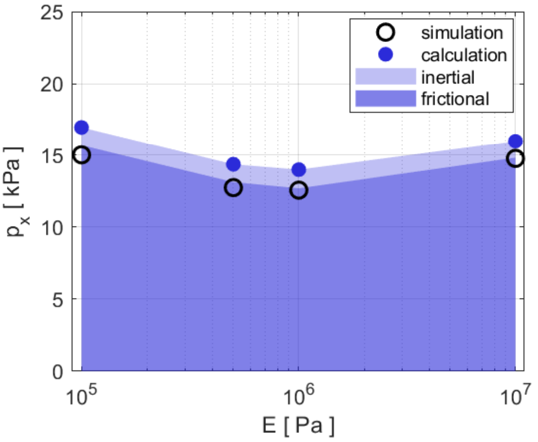

Fig. S.4 Comparison of simulated and estimated impact pressure impact pressure p_x on a cylindrical obstacle with $w = 1$ m impacted by granular plug flows at 3 m/s with varying E.

Figure S.4 shows that even for varying E the pressure can be estimated with an average relative error of 11 %. The error may come from the fact that we simulated a shallow flow with $h = 1$ m in this study. Distortions at the bottom wall and the free surface therefore had

a larger influence on the overall accuracy. Nevertheless, the calculated pressure agrees well with the simulated impact pressure and we concluded that the established link between the MD properties and the impact pressure did not depend significantly on E.

S.3 **Sensitivity analysis of axial compression tests**

A large portion of the main article's results depend on the relationship between the relative particle interpenetration Δ and the normal stress σ_n obtained from compression tests (section 2.4). To assess the robustness of the compression tests' results, we run a number of compression tests varying the sample size s_0, the compression speed and restitution coefficient e_r. In Figure S.5 we present the results of this sensitivity analysis.

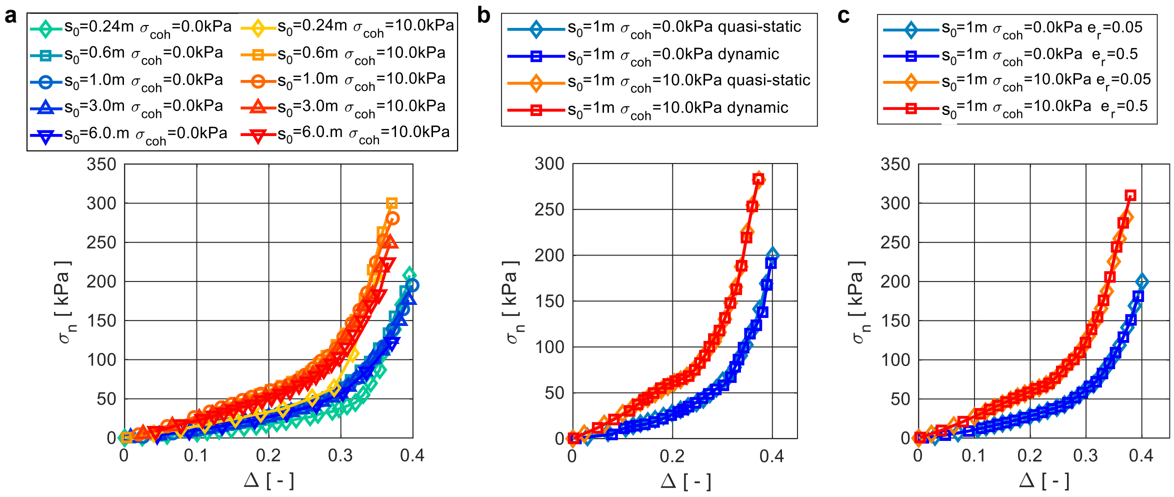

Fig. S.5 Sensitivity analysis for the axial compression tests with varying sample size (a), compression speed (b) and restitution coefficient (c).

- **Sample size:** We vary the size of the compression sample in the range of our obstacle widths 0.24 m $\leq w \leq$ 6 m. Figure S.5 a shows that, relative to the largest sample with $s_0 = 6$ m in the cohesionless case, the normal stress σ_n deviates by 25.3 %, 8.2 %, 7.3 % and 2.9 % for the samples with s_0 of 0.24 m, 0.6 m, 1.0 m and 3.0 m, respectively. Similarly, the results of the compression tests with the cohesive material also converge towards the results obtained with $s_0 = 6$ m.

- **Compression speed:** The compression test reported in the main manuscript corresponds to a *quasi-static* compression test. In these tests we compress the sample by a small increment and run the simulation until the material reaches a mechanical equilibrium criterion. This process is repeated until the target strain is reached. In the *dynamic* test we compress the sample by moving the upper clump (see Figure 3 in the main manuscript) at a constant speed of 3 m/s. This corresponds to the approach speed $v = 3$ m/s of the flow used in our simulations.
  Hence, to assess the influence of the strain rate, in Figure S.5 b we compare the stresses of a quasi-static and a dynamic compression test. The results of the *quasi-static* and *dynamic* compression tests differ by 3.4 % and 0.5 % in the cohesionless and the cohesive case, respectively.

– **Restitution coefficient:** In this study we choose e_r in accordance with our earlier study [1], where we choose a low damping $e_r = 0.05$ (section 2.3). In order to estimate the influence of this choice on the resulting impact pressure, we perform compression tests of the granular material, as described in the article and varying e_r of the cohesionless material, where we expect a stronger influence of e_r. Figure S.5 c below shows that we do not expect significant differences in the impact pressure even if we greatly increase e_r to $e_r = 0.5$, as the stresses within the material, on the wall and on the clump, are practically identical. The differences between the two cases are < 0.5 % and are therefore negligible.

S.4 Definition of the mobilized domain (MD)

As described in the main article, we use a percentile threshold of the contact forces between the particles to systematically identify the mobilized domain (MD). Even in an undisturbed granular bed the contact forces increase analogous to a hydrostatic pressure for increasing depth from the free surface. Hence, we calculate the threshold for every height increment of the discretized flow field in the vertical direction individually.
The percentile threshold we use for the MD identification is only consistent if the ratio of particles inside and outside the MD is similar for all simulations. However, in our simulations we vary the obstacle size (0.24 m $\leq w \leq 6$ m) and use two different flow domain widths ($D_y = 11$ m, $D_y = 22$ m). This leads to different proportions of particles inside and outside of the MD. In order to improve the consistency of the MD threshold values between all simulations, we must keep the ratio between particles inside and outside of the MD approximately constant. Therefore, for the MD threshold calculation in simulations where $w < 6$ m we consider only a smaller part of the flow domain proportional to w, which scales with MD size. We visualize the flow domain and the domain considered for the MD threshold calculation schematically in a horizontal section in Figure S.6. The considered domain is limited by the simulation of the 6 m-wide cylindrical obstacle, where the simulation domain around the obstacle is the smallest compared with the extent of the obstacle itself. For the identification of the MD itself, the threshold value is applied to the whole flow domain.
Moreover, we use a Gaussian filter to smooth the contours of the MD.

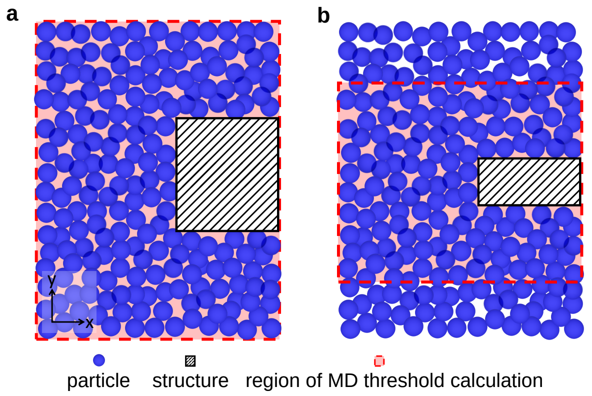

Fig. S.6 Schematic drawing of a horizontal cross-section through the flow domain for a wide (a) obstacle (black and white hatched area) and a narrow (b) obstacle. The domain considered for the MD threshold calculation is shaded in red and outlined with a red dashed line. The flow domain, the particles and the obstacle are not to scale.

S.5 **Sensitivity of the results on the MD threshold value**

In this study the definition of the MD is crucial. Hence, we perform a sensitivity analysis on the percentile threshold we use to identify the MD. There are the two extreme cases. For the 0th percentile the whole flow domain is considered as the MD. For the case of the 100th percentile the MD vanishes altogether. Hence, a physically relevant threshold value has to be chosen in between these two values.
In order to evaluate how much our results vary if we change the threshold for the MD calculation, we perform a sensitivity analysis. Figure S.7 a and b show the streamwise and transverse extent of the identified MD normalized by the respective flow domain size as a function of the threshold. Figure S.7 c shows the relative error between the simulated impact pressure values, which are independent of the MD definition, and the estimated impact pressure values (sections 3.2 and 3.3 of the main article), which are based on the properties of the identified domain.

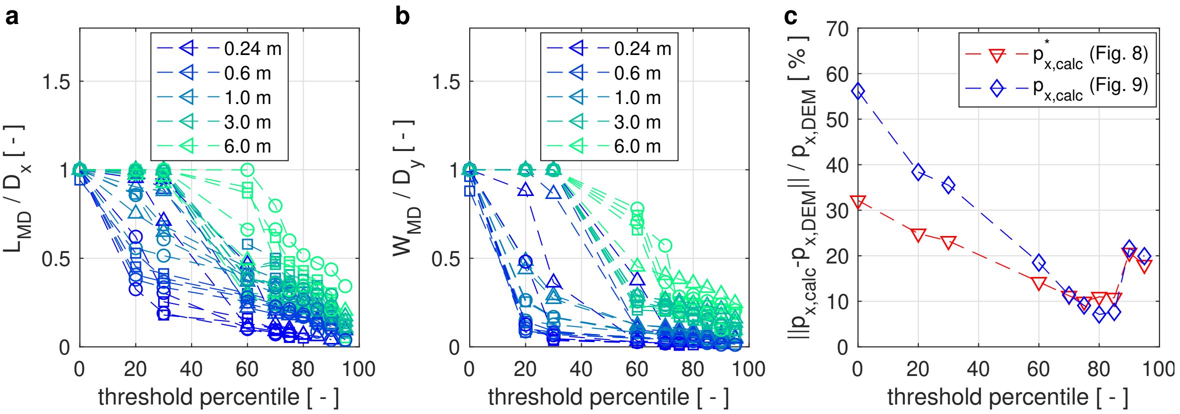

Fig. S.7 Sensitivity of the results on the MD threshold value. The MD length L_{MD} (a), the MD width W_{MD} (b) and the relative errors of the calculated pressures $p_{x,calc}, p^*_{x,calc}$ (c) are shown for different MD thresholds. The color in the left and the middle panel corresponds to the obstacle width.

Figure S.7 a and b show, that for thresholds below the 30th percentile in most simulations, the whole flow domain is considered the MD. For thresholds above 95th percentile the MD is almost vanishing in most simulations. Figure S.7 c confirms that the relative errors are highest for extremely low and extremely high threshold values. For the values higher than 95th percentile we cannot calculate the error because the MD vanishes in most simulations altogether. Both curves of relative error for the estimation of the cohesionless $p_{x,calc}$ and the cohesive pressure $p^*_{x,calc}$ have a robust minimum around a threshold value of 80th percentile. Hence, varying the threshold value between the 70th percentile and the 90th percentile does not influence the outcome of our results considerably. The change in the extent of the MD in these bounds of the threshold is visualized in Figure S.8, analogous to Figure 4 b in the main article.

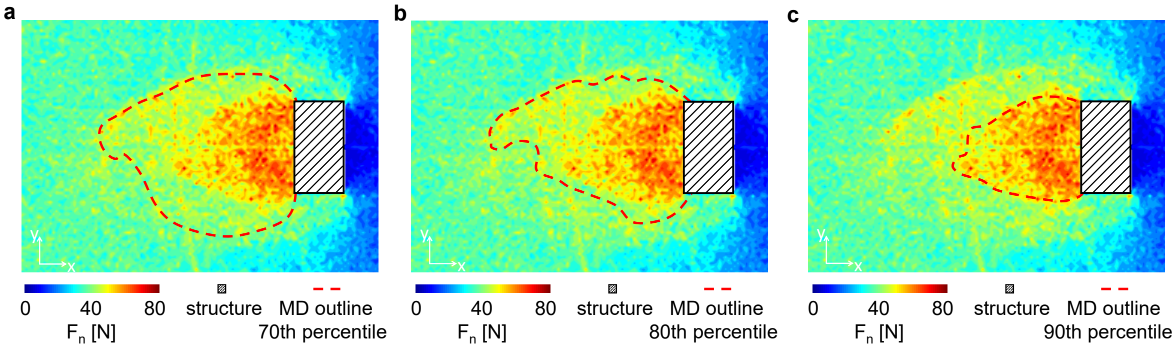

Fig. S.8 Sensitivity of the MD extent on the MD threshold value. Panels a, b and c show the MD extent for threshold values of 70th, 80th and 90th percentile, respectively.

S.6 Impact pressure calculated from reported drag forces in the literature

Existing literature on the interaction of granular materials and immersed intruders moving relative to each other often report the drag force exerted on the intruder by the granular material. In our main article we are mostly concerned with the average impact pressure on an obstacle exerted by the granular flow. We find that the average impact pressure decreases non-linearly for increasing obstacle widths. Although it seems counterintuitive at first, this trend is known to be true for granular snow avalanches [10]. In order to find out whether this behavior is common for various configurations of granular materials interacting with intruders, we recalculate the reported drag force in the existing literature [11,12,13] as the average impact pressure and plot them as a function of the intruder's characteristic size in Figure S.9. The results in Figure S.9 clearly confirm the trend of decreasing impact pressure for intruders of increasing width.

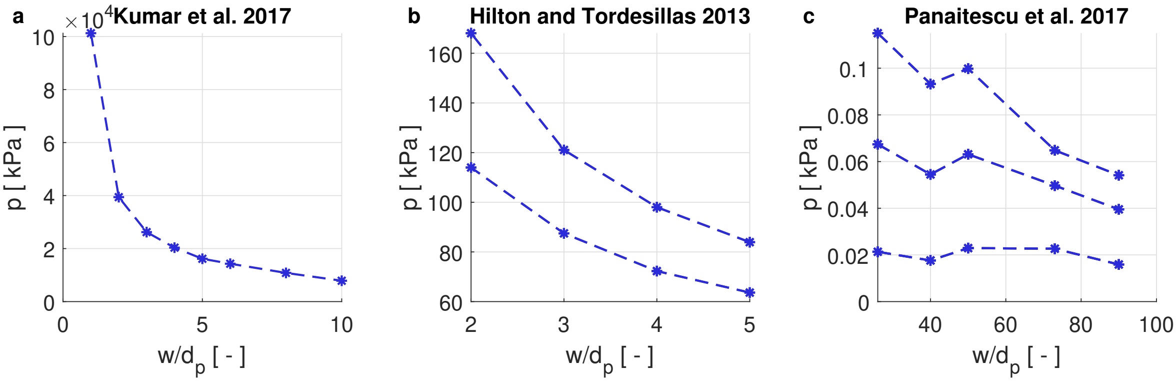

Fig. S.9 Impact pressure calculated from reported drag forces from the literature. Panels a, b and c show the data from [11], [12] and [13], respectively.

S.7 Impact pressure scaling law for cohesive avalanches

In an earlier paper [1], we found that the impact pressure increase due to cohesion depends on the competition of cohesive and inertial forces of the impacting granular flow. Hence, according to Eq. (1) the pressure exerted by a cohesionless flow with the same flow height and velocity $p_{x,calc}$ can be multiplied by a factor $f_{coh}(q_{Bo,Fr})$ to obtain the impact pressure $p^*_{x,calc}$ of an equivalent but cohesive flow. For a specific obstacle geometry the factor $f_{coh}(q_{Bo,Fr})$

is a unique function of the ratio of the Bond and the Froude number $q_{Bo,Fr} = Bo/Fr$ [1]. The dimensionless Bond number $Bo = \sigma_{coh}/p_{conf}$ is the cohesive strength σ_{coh} divided by the confining pressure p_{conf} [14], which is the vertical component of the local stress tensor.

$$p^*_{x,calc} = p_{x,calc}\ f_{coh}(q_{Bo,Fr}) \tag{1}$$

The results from [1] are specific for the geometry of an existing measurement structure at the "Vallée de la Sionne" avalanche test site in Valais, Switzerland. While we assume that f_{coh} varies depending on the obstacle geometry, f_{coh} could be obtained for other structure geometries by performing a number of simulations in which the granular flow velocity and cohesion are varied. Because this is a computationally intensive undertaking, we use the values found in [1] as an approximation.

References

1. Kyburz M.L., Sovilla B., Gaume J., Ancey C.: Decoupling the role of inertia, friction, and cohesion in dense granular avalanche pressure build-up on obstacles. J. Geophys. Res. Earth. Surf. 125(2), e2019JF005192 (2020). https://doi.org/10.1029/2019JF005192
2. Bucher E., Roch A., Reibungs- und Packungswiederstände bei raschen Schneebewegungen. Tech. Rep. 31 SLF (1946)
3. Voellmy A.: Über die Zerstörungskraft von Lawinen. III. Stau- und Druckwirkungen. Schweizerische Bauzeitung 73, 246 (1955)
4. Kuroiwa D., Mizuno Y., Takeuchi M.: Micromeritical properties of snow. Physics of Snow and Ice: proceedings 1(2), 751 (1967)
5. Yong R., Fukue M.: Performance of snow in confined compression. Journal of Terramechanics 14(2), 59 (1977). https://doi.org/10.1016/0022-4898(77)90002-7
6. Shapiro L.H., Johnson J., Sturm M., Blaisdell G.: Snow mechanics: Review of the state of knowledge and applications. CRREL Report 97-3 p. 40 (1997)
7. Scapozza C., Entwicklung eines dichte-und temperaturabhängigen Stoffgesetzes zur Beschreibung des visko-elastischen Verhaltens von Schnee. Phdthesis ETH Zürich (2004). https://doi.org/10.3929/ethz-a-004680249
8. Gaume J., van Herwijnen A., Chambon G., Birkeland K.W., Schweizer J.: Modeling of crack propagation in weak snowpack layers using the discrete element method. The Cryosphere 9(5), 1915 (2015). https://doi.org/10.5194/tc-9-1915-2015
9. Gerling B., Löwe H., van Herwijnen A.: Measuring the Elastic Modulus of Snow. Geophys. Res. Lett. 44(21), 11,088 (2017). https://doi.org/10.1002/2017GL075110
10. Margreth S.: Snow pressure on cableway masts: Analysis of damages and design approach. Cold Reg. Sci. Tech. 47(1), 4 (2007). https://doi.org/10.1016/j.coldregions.2006.08.020
11. Kumar S., Anki Reddy K., Takada S., Hayakawa H.: Scaling law of the drag force in dense granular media. arXiv e-prints arXiv:1712.09057 (2017)
12. Hilton J.E., Tordesillas A.: Drag force on a spherical intruder in a granular bed at low froude number. Phys. Rev. E 88, 062203 (2013). https://doi.org/10.1103/PhysRevE.88.062203
13. Panaitescu A., Clotet X., Kudrolli A.: Drag law for an intruder in granular sediments. Phys. Rev. E 95, 032901 (2017). https://doi.org/10.1103/PhysRevE.95.032901
14. Roy S., Luding S., Weinhart T.: A general(ized) local rheology for wet granular materials. New Journal of Physics 19(4), 043014 (2017). https://doi.org/10.1088/1367-2630/aa6141
